# 7-*tert*-Butyl-1,3-bis­(eth­oxy­meth­yl)pyrene

**DOI:** 10.1107/S241431462600413X

**Published:** 2026-04-29

**Authors:** Tetsuji Moriguchi, Misa Sasaki, Noriko Miyoshi

**Affiliations:** a Department of Material Science, Faculty of Engineering, Kyushu Institute of Technology, 1-1 Sensui-cho, Tobata-ku Kitakyushu, Fukuoka, Japan; b Technical Support Department, Management Headquarters, Kyushu Institute of Technology, 1-1 Sensui-cho, Tobata-ku, Kitakyushu 804-8550, Japan; Vienna University of Technology, Austria

**Keywords:** crystal structure, substituted pyrene derivative, π–π inter­actions

## Abstract

In the mol­ecular structure of the title compound, C_26_H_30_O_2_, the two eth­oxy­methyl side arms are twisted from the almost planar pyrene system by 29.85 (17) and 15.16 (18)°. In the crystal, adjacent mol­ecules are arranged in pairs that mainly inter­act through π–π inter­actions.

## Abstract 2

Two mol­ecules of 7-*tert*-butyl-1,3-bis­(eth­oxy­meth­yl)pyrene form a dimer unit through π–π inter­actions in the crystal.

## Graphical abstract

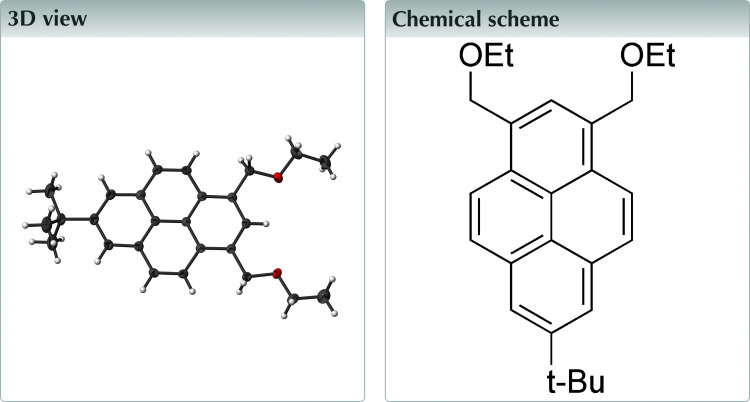

## Structure description

In recent years, polycyclic aromatic hydro­carbons (PAHs) have been attracting great inter­est owing to their significant photochemical and electrical properties (Dötz *et al.*, 2000 ▸). In the PAHs, pyrene and its derivatives are probably the most studied compounds. They constitute an important class of PAHs found in charcoal and are valuable as inter­mediates. Moreover, the PAHs comprising the pyrene moiety exhibit *p*-type semiconductor properties (Moriguchi *et al.*, 2017 ▸). In the context of our previous studies with respect to substituted pyrene derivatives (Moriguchi *et al.*, 2015 ▸; Moriguchi *et al.*, 2018 ▸) or a lanthanum complex with four pyrene moieties (Moriguchi *et al.*, 2014 ▸), in order to evaluate its fluorescence properties, we report here the result of the crystal structure analysis of 7-*tert*-butyl-1,3-bis­(eth­oxy­meth­yl)pyrene.

**Figure 1:**
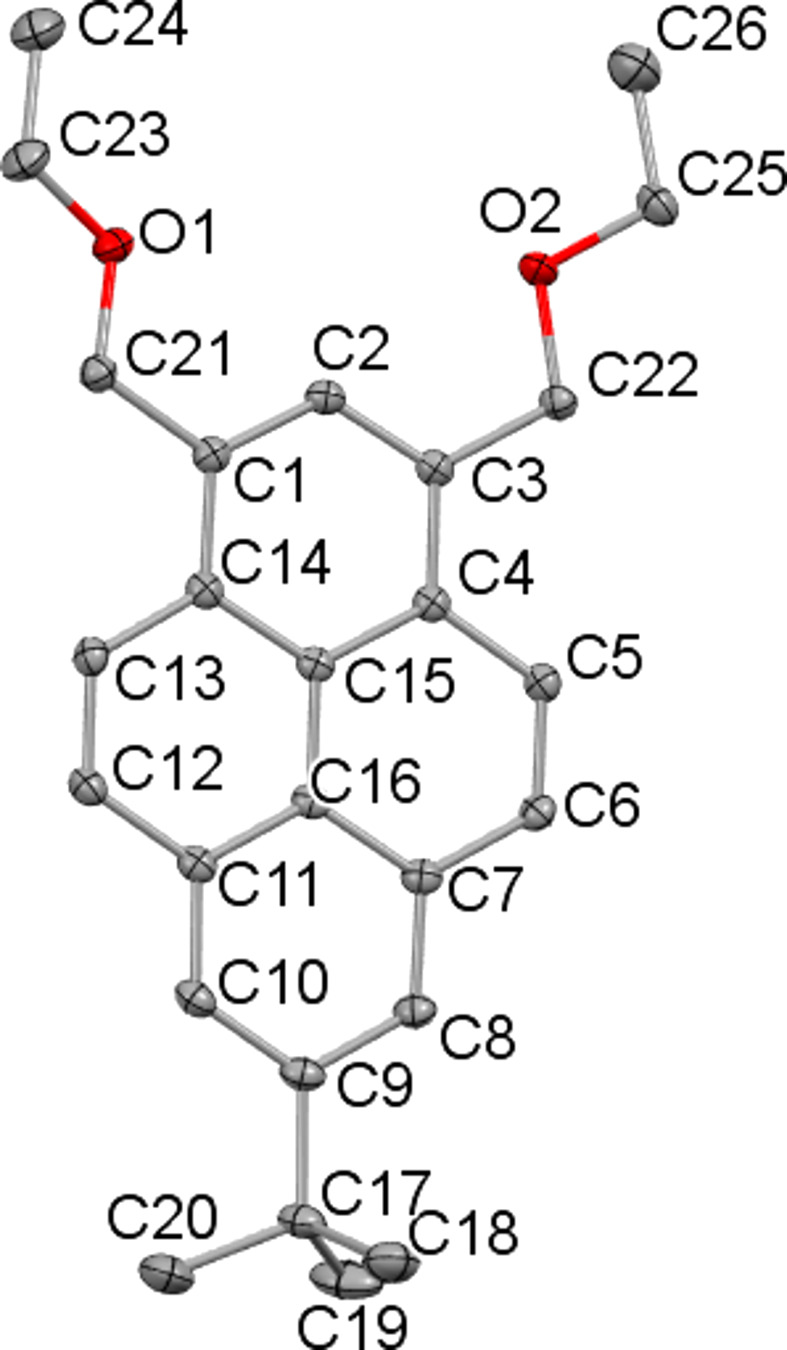
The mol­ecular structure and atom-numbering scheme for the title compound, with displacement ellipsoids drawn at the 50% probability level. H atoms have been omitted for clarity.

The mol­ecular structure is shown in Fig. 1 ▸. The pyrene ring system is almost planar, with an r.m.s. deviation of the non-H atoms of 0.011 Å. The dihedral angles between the planes of the pyrene system and the eth­oxy­methyl groups are 29.85 (17) (C21/O1/C23/C24) and 15.16 (18)° (C22/O2/C25/C26).

**Figure 2:**
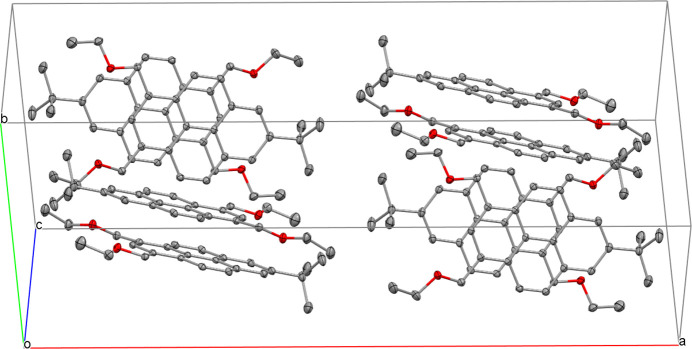
Packing diagram of the title compound, with displacement ellipsoids drawn at the 50% probability level. H atoms have been omitted for clarity.

**Figure 3:**
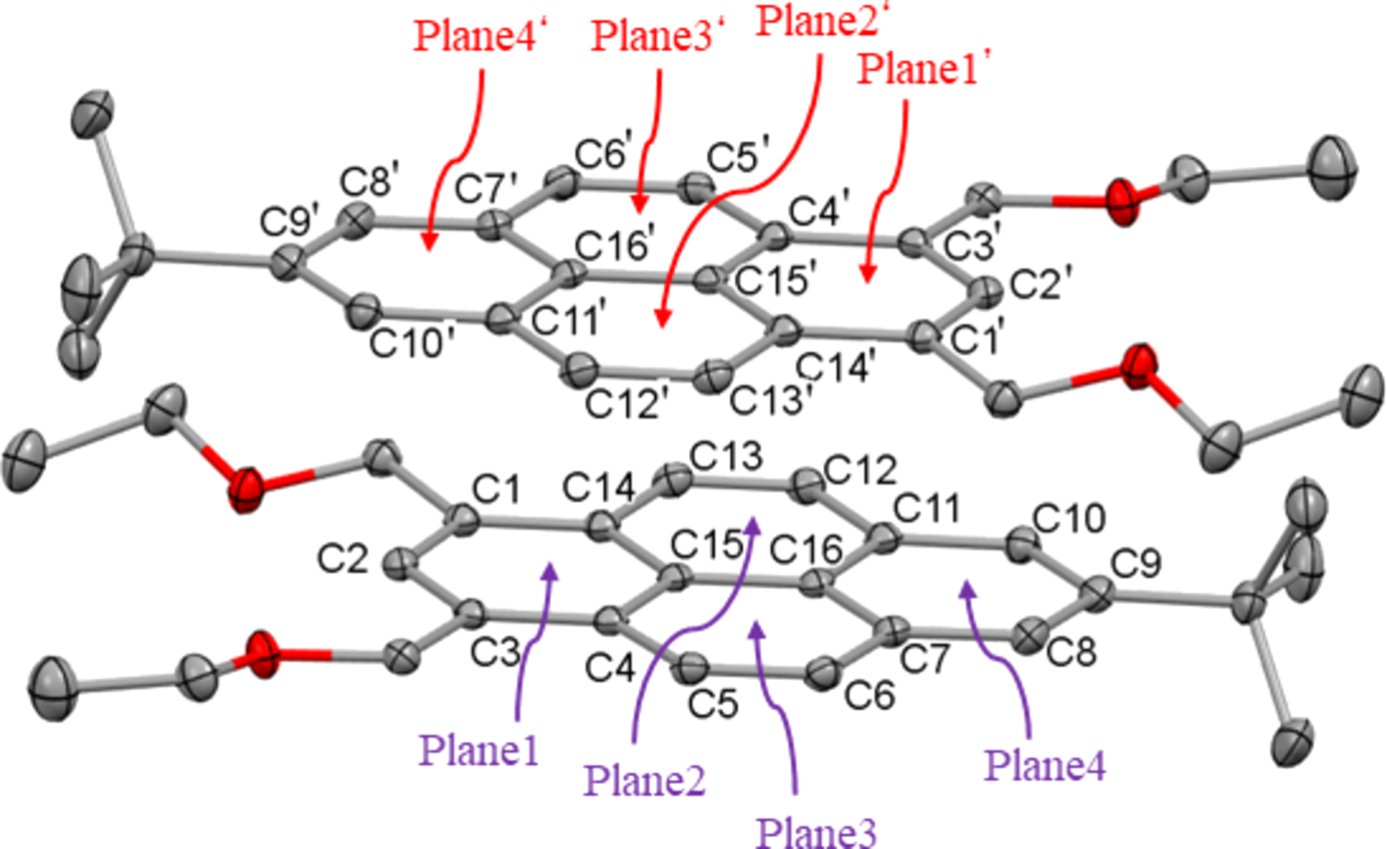
A pair of mol­ecules linked by inter­molecular π–π inter­actions and definitions of the planes. The mol­ecule with primed labels is related by symmetry code (−*x* + 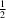, −*y* + 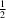, −*z*).

The packing of the mol­ecules within the unit cell is shown in Fig. 2 ▸. A particular arrangement results from π–π inter­actions between mol­ecules arranged in pairs (Fig. 3 ▸), with centroid-to-centroid distances of *Cg*1⋯*Cg*3′ of 3.6216 (8) Å (slippage 1.115 Å), *Cg*1⋯*Cg*2′ of 3.9104 (8) Å (slippage 1.840 Å), *Cg*1⋯*Cg*4′ of 3.7156 (8) Å (slippage 1.408 Å) and *Cg*3⋯*Cg*2′ of 3.7092 (8) Å (slippage 1.403 Å) [*Cg*1 is the centroid of ring C1–C4/C14/C15 (plane 1), *Cg*2 is the centroid of ring C11–C16 (plane 2), *Cg*3 is the centroid of ring C4–C7/C15/C16 (plane 3) and *Cg*4 is the centroid of ring C7–C11/C16 (plane 4); symmetry code for primed centroids: −*x* + 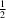, −*y* + 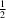, −*z*]. These values are approximately equal or smaller than the sum of the van der Waals radii of aromatic planes (Rowland & Taylor, 1996 ▸).

## Synthesis and crystallization

A tetra­hydro­furan solution (50 ml) of 4-*tert*-butyl-1,3-bis­(chloro­meth­yl)pyrene (0.10 mmol) was added dropwise to ethanol (1 mmol) in the presence of an excess of sodium (0.5 mmol). The mixed solution was then stirred for 3 h at room temperature before the volatiles were removed under reduced pressure. The crude reaction mixture was subjected to column chromatography using EtOAc/hexane as the mobile phase. The title compound was isolated as a yellow fluorescent solid with 80% yield. Single crystals were obtained by the vapour diffusion method at room temperature by diffusion of hexane into a chloro­form solution. MS: *M*^+^, 374.

## Refinement

Crystal data, data collection and structure refinement details are summarized in Table 1 ▸.

## Supplementary Material

Crystal structure: contains datablock(s) I. DOI: 10.1107/S241431462600413X/wm4247sup1.cif

Structure factors: contains datablock(s) I. DOI: 10.1107/S241431462600413X/wm4247Isup2.hkl

Supporting information file. DOI: 10.1107/S241431462600413X/wm4247Isup3.cml

CCDC reference: 2547421

Additional supporting information:  crystallographic information; 3D view; checkCIF report

Additional supporting information:  crystallographic information; 3D view; checkCIF report

## Figures and Tables

**Table 1:** Experimental details

Crystal data	
Chemical formula	C_26_H_30_O_2_
*M*_r_	374.50
Crystal system, space group	Monoclinic, *C*2/*c*
Temperature (K)	90
*a*, *b*, *c* (Å)	31.563 (2), 13.4849 (10), 9.4170 (7)
β (°)	92.464 (1)
*V* (Å^3^)	4004.4 (5)
*Z*	8
Radiation type	Mo *K*α
μ (mm^−1^)	0.08
Crystal size (mm)	0.50 × 0.35 × 0.25

Data collection	
Diffractometer	Bruker APEXII CCD
Absorption correction	Multi-scan (*SADABS*; Krause *et al.*, 2015 ▸)
*T*_min_, *T*_max_	0.666, 0.746
No. of measured, independent and observed [*I* > 2σ(*I*)] reflections	23061, 4876, 3900
*R*_int_	0.031
(sin θ/λ)_max_ (Å^−1^)	0.676

Refinement	
*R*[*F*^2^ > 2σ(*F*^2^)], *wR*(*F*^2^), *S*	0.055, 0.173, 1.05
No. of reflections	4876
No. of parameters	258
H-atom treatment	H-atom parameters constrained
Δρ_max_, Δρ_min_ (e Å^−3^)	0.46, −0.47

Computer programs: *SMART* and *SAINT* (Bruker, 2009 ▸), *SHELXS* (Sheldrick, 2008 ▸), *SHELXL* (Sheldrick, 2015 ▸), *OLEX2* (Dolomanov *et al.*, 2009 ▸) and *publCIF* (Westrip, 2010 ▸).

## full crystallographic data

### 7-tert-Butyl-1,3-bis(ethoxymethyl)pyrene Crystal data

**Table:**

C_26_H_30_O_2_	*F*(000) = 1616
*M**_r_* = 374.50	*D*_x_ = 1.242 Mg m^−^^3^
Monoclinic, *C*2/*c*	Mo *K*α radiation, λ = 0.71073 Å
*a* = 31.563 (2) Å	Cell parameters from 7217 reflections
*b* = 13.4849 (10) Å	θ = 2.5–28.5°
*c* = 9.4170 (7) Å	µ = 0.08 mm^−^^1^
β = 92.464 (1)°	*T* = 90 K
*V* = 4004.4 (5) Å^3^	Prism, clear light colourless
*Z* = 8	0.50 × 0.35 × 0.25 mm

### 7-tert-Butyl-1,3-bis(ethoxymethyl)pyrene Data collection

**Table:**

Bruker APEXII CCD diffractometer	4876 independent reflections
Radiation source: sealed tube	3900 reflections with *I* > 2σ(*I*)
Graphite monochromator	*R*_int_ = 0.031
Detector resolution: 8 pixels mm^-1^	θ_max_ = 28.7°, θ_min_ = 1.6°
φ and ω scans	*h* = −40→41
Absorption correction: multi-scan (*SADABS*; Krause *et al.*, 2015)	*k* = −17→18
*T*_min_ = 0.666, *T*_max_ = 0.746	*l* = −12→12
23061 measured reflections	

### 7-tert-Butyl-1,3-bis(ethoxymethyl)pyrene Refinement

**Table:**

Refinement on *F*^2^	Primary atom site location: structure-invariant direct methods
Least-squares matrix: full	Hydrogen site location: inferred from neighbouring sites
*R*[*F*^2^ > 2σ(*F*^2^)] = 0.055	H-atom parameters constrained
*wR*(*F*^2^) = 0.173	*w* = 1/[σ^2^(*F*_o_^2^) + (0.0977*P*)^2^ + 3.0623*P*] where *P* = (*F*_o_^2^ + 2*F*_c_^2^)/3
*S* = 1.05	(Δ/σ)_max_ < 0.001
4876 reflections	Δρ_max_ = 0.46 e Å^−^^3^
258 parameters	Δρ_min_ = −0.47 e Å^−^^3^
0 restraints	

### 7-tert-Butyl-1,3-bis(ethoxymethyl)pyrene Special details

**Table:**

Geometry. All esds (except the esd in the dihedral angle between two l.s. planes) are estimated using the full covariance matrix. The cell esds are taken into account individually in the estimation of esds in distances, angles and torsion angles; correlations between esds in cell parameters are only used when they are defined by crystal symmetry. An approximate (isotropic) treatment of cell esds is used for estimating esds involving l.s. planes.

### 7-tert-Butyl-1,3-bis(ethoxymethyl)pyrene Fractional atomic coordinates and isotropic or equivalent isotropic displacement parameters (Å^2^)

**Table:**

	*x*	*y*	*z*	*U*_iso_*/*U*_eq_	
O1	0.12093 (3)	0.49719 (8)	0.09130 (12)	0.0282 (3)	
O2	0.14358 (3)	0.23373 (8)	0.39513 (11)	0.0264 (3)	
C1	0.18957 (4)	0.41990 (10)	0.09130 (14)	0.0202 (3)	
C2	0.17553 (4)	0.35367 (10)	0.19234 (14)	0.0200 (3)	
H2	0.147300	0.356026	0.216684	0.024*	
C3	0.20235 (4)	0.28392 (10)	0.25827 (13)	0.0189 (3)	
C4	0.24518 (4)	0.27928 (10)	0.22227 (13)	0.0184 (3)	
C5	0.27447 (4)	0.20736 (10)	0.28424 (14)	0.0207 (3)	
H5	0.264937	0.162385	0.350582	0.025*	
C6	0.31544 (4)	0.20404 (11)	0.24785 (14)	0.0221 (3)	
H6	0.333469	0.156872	0.289762	0.026*	
C7	0.33165 (4)	0.27162 (10)	0.14618 (14)	0.0208 (3)	
C8	0.37391 (4)	0.26903 (11)	0.10699 (15)	0.0242 (3)	
H8	0.392067	0.222113	0.148895	0.029*	
C9	0.38982 (4)	0.33436 (11)	0.00726 (15)	0.0249 (3)	
C10	0.36178 (4)	0.40291 (11)	−0.05576 (15)	0.0237 (3)	
H10	0.371724	0.446394	−0.123375	0.028*	
C11	0.31912 (4)	0.40857 (10)	−0.02072 (14)	0.0208 (3)	
C12	0.29017 (4)	0.47819 (11)	−0.08668 (15)	0.0230 (3)	
H12	0.299875	0.521467	−0.154981	0.028*	
C13	0.24900 (4)	0.48248 (10)	−0.05191 (15)	0.0220 (3)	
H13	0.231062	0.528372	−0.097248	0.026*	
C14	0.23239 (4)	0.41764 (10)	0.05358 (14)	0.0197 (3)	
C15	0.26024 (4)	0.34700 (10)	0.12030 (13)	0.0180 (3)	
C16	0.30358 (4)	0.34268 (10)	0.08249 (14)	0.0190 (3)	
C17	0.43655 (5)	0.32752 (13)	−0.02992 (17)	0.0297 (3)	
C18	0.44405 (6)	0.22943 (15)	−0.1055 (2)	0.0440 (5)	
H18A	0.436099	0.175498	−0.045671	0.066*	
H18B	0.473517	0.223630	−0.125507	0.066*	
H18C	0.427258	0.227436	−0.192908	0.066*	
C19	0.46469 (6)	0.3341 (2)	0.1055 (2)	0.0526 (6)	
H19A	0.458905	0.394888	0.154212	0.079*	
H19B	0.493939	0.332769	0.081555	0.079*	
H19C	0.458934	0.278932	0.166122	0.079*	
C20	0.44954 (6)	0.41281 (16)	−0.1274 (2)	0.0488 (5)	
H20A	0.432821	0.410135	−0.214909	0.073*	
H20B	0.479018	0.406459	−0.146977	0.073*	
H20C	0.444898	0.475073	−0.081145	0.073*	
C21	0.15861 (4)	0.48931 (11)	0.01536 (15)	0.0236 (3)	
H21A	0.171421	0.554309	0.006909	0.028*	
H21B	0.151861	0.464552	−0.079645	0.028*	
C22	0.18585 (4)	0.21118 (10)	0.36365 (14)	0.0211 (3)	
H22A	0.187148	0.144655	0.324899	0.025*	
H22B	0.203585	0.213168	0.450330	0.025*	
C23	0.08765 (5)	0.54219 (14)	0.0076 (2)	0.0377 (4)	
H23A	0.079379	0.499682	−0.071942	0.045*	
H23B	0.097029	0.605225	−0.029458	0.045*	
C24	0.05040 (6)	0.55790 (16)	0.1017 (2)	0.0463 (5)	
H24A	0.027562	0.588779	0.047480	0.069*	
H24B	0.058927	0.599810	0.180190	0.069*	
H24C	0.041134	0.495061	0.136829	0.069*	
C25	0.12618 (5)	0.15790 (12)	0.47949 (17)	0.0310 (3)	
H25A	0.144671	0.146157	0.562741	0.037*	
H25B	0.124111	0.096805	0.425257	0.037*	
C26	0.08382 (6)	0.18715 (17)	0.5240 (2)	0.0510 (5)	
H26A	0.065729	0.199860	0.441426	0.076*	
H26B	0.086119	0.246069	0.581013	0.076*	
H26C	0.071990	0.134609	0.578500	0.076*	

### 7-tert-Butyl-1,3-bis(ethoxymethyl)pyrene Atomic displacement parameters (Å^2^)

**Table:**

	*U*^11^	*U*^22^	*U*^33^	*U*^12^	*U*^13^	*U*^23^
O1	0.0200 (5)	0.0328 (6)	0.0319 (6)	0.0042 (4)	0.0009 (4)	0.0053 (4)
O2	0.0232 (5)	0.0280 (5)	0.0286 (5)	0.0012 (4)	0.0092 (4)	0.0056 (4)
C1	0.0207 (6)	0.0205 (6)	0.0191 (6)	−0.0004 (5)	−0.0011 (5)	−0.0030 (5)
C2	0.0186 (6)	0.0229 (7)	0.0186 (6)	−0.0017 (5)	0.0005 (5)	−0.0035 (5)
C3	0.0210 (6)	0.0207 (6)	0.0150 (6)	−0.0024 (5)	0.0010 (5)	−0.0035 (5)
C4	0.0204 (6)	0.0195 (6)	0.0152 (6)	−0.0018 (5)	−0.0014 (5)	−0.0036 (5)
C5	0.0231 (7)	0.0229 (6)	0.0160 (6)	−0.0017 (5)	−0.0012 (5)	−0.0002 (5)
C6	0.0213 (6)	0.0245 (7)	0.0199 (6)	0.0011 (5)	−0.0034 (5)	0.0002 (5)
C7	0.0194 (6)	0.0254 (7)	0.0174 (6)	−0.0019 (5)	−0.0017 (5)	−0.0036 (5)
C8	0.0193 (7)	0.0293 (7)	0.0238 (7)	−0.0002 (5)	−0.0022 (5)	−0.0016 (6)
C9	0.0195 (6)	0.0314 (7)	0.0238 (7)	−0.0043 (6)	0.0010 (5)	−0.0044 (6)
C10	0.0219 (7)	0.0280 (7)	0.0213 (7)	−0.0066 (5)	0.0012 (5)	−0.0003 (5)
C11	0.0215 (7)	0.0234 (7)	0.0173 (6)	−0.0047 (5)	−0.0007 (5)	−0.0030 (5)
C12	0.0239 (7)	0.0250 (7)	0.0199 (6)	−0.0054 (5)	−0.0002 (5)	0.0020 (5)
C13	0.0235 (7)	0.0219 (6)	0.0202 (6)	−0.0014 (5)	−0.0022 (5)	0.0012 (5)
C14	0.0211 (6)	0.0205 (6)	0.0173 (6)	−0.0026 (5)	−0.0012 (5)	−0.0027 (5)
C15	0.0189 (6)	0.0201 (6)	0.0148 (6)	−0.0032 (5)	−0.0012 (5)	−0.0043 (5)
C16	0.0186 (6)	0.0217 (6)	0.0166 (6)	−0.0034 (5)	−0.0014 (5)	−0.0047 (5)
C17	0.0181 (7)	0.0408 (9)	0.0304 (8)	−0.0033 (6)	0.0023 (6)	0.0003 (7)
C18	0.0296 (9)	0.0502 (11)	0.0529 (11)	0.0012 (8)	0.0112 (8)	−0.0052 (9)
C19	0.0233 (8)	0.0935 (17)	0.0408 (10)	−0.0099 (9)	−0.0019 (7)	−0.0037 (10)
C20	0.0279 (9)	0.0553 (12)	0.0643 (13)	−0.0033 (8)	0.0137 (8)	0.0125 (10)
C21	0.0222 (7)	0.0249 (7)	0.0238 (7)	0.0007 (5)	0.0006 (5)	0.0009 (5)
C22	0.0212 (7)	0.0233 (6)	0.0190 (6)	0.0005 (5)	0.0017 (5)	−0.0007 (5)
C23	0.0278 (8)	0.0406 (9)	0.0440 (10)	0.0084 (7)	−0.0060 (7)	0.0047 (7)
C24	0.0290 (9)	0.0485 (11)	0.0613 (13)	0.0075 (8)	−0.0006 (8)	0.0051 (9)
C25	0.0322 (8)	0.0314 (8)	0.0297 (8)	−0.0051 (6)	0.0063 (6)	0.0058 (6)
C26	0.0440 (11)	0.0556 (12)	0.0543 (12)	0.0011 (9)	0.0127 (9)	0.0115 (10)

### 7-tert-Butyl-1,3-bis(ethoxymethyl)pyrene Geometric parameters (Å, º)

**Table:**

O1—C21	1.4177 (17)	C14—C15	1.4239 (19)
O1—C23	1.4219 (18)	C15—C16	1.4295 (18)
O2—C22	1.4123 (16)	C17—C18	1.525 (3)
O2—C25	1.4198 (18)	C17—C19	1.525 (2)
C1—C2	1.3912 (19)	C17—C20	1.538 (2)
C1—C14	1.4126 (19)	C18—H18A	0.9600
C1—C21	1.5107 (19)	C18—H18B	0.9600
C2—H2	0.9300	C18—H18C	0.9600
C2—C3	1.3935 (19)	C19—H19A	0.9600
C3—C4	1.4089 (18)	C19—H19B	0.9600
C3—C22	1.5042 (18)	C19—H19C	0.9600
C4—C5	1.4455 (19)	C20—H20A	0.9600
C4—C15	1.4217 (18)	C20—H20B	0.9600
C5—H5	0.9300	C20—H20C	0.9600
C5—C6	1.3527 (19)	C21—H21A	0.9700
C6—H6	0.9300	C21—H21B	0.9700
C6—C7	1.4321 (19)	C22—H22A	0.9700
C7—C8	1.3998 (19)	C22—H22B	0.9700
C7—C16	1.4201 (19)	C23—H23A	0.9700
C8—H8	0.9300	C23—H23B	0.9700
C8—C9	1.397 (2)	C23—C24	1.517 (3)
C9—C10	1.395 (2)	C24—H24A	0.9600
C9—C17	1.533 (2)	C24—H24B	0.9600
C10—H10	0.9300	C24—H24C	0.9600
C10—C11	1.4021 (19)	C25—H25A	0.9700
C11—C12	1.433 (2)	C25—H25B	0.9700
C11—C16	1.4196 (19)	C25—C26	1.472 (2)
C12—H12	0.9300	C26—H26A	0.9600
C12—C13	1.3550 (19)	C26—H26B	0.9600
C13—H13	0.9300	C26—H26C	0.9600
C13—C14	1.4390 (19)		
			
C21—O1—C23	111.60 (12)	C19—C17—C20	107.14 (15)
C22—O2—C25	110.73 (11)	C17—C18—H18A	109.5
C2—C1—C14	119.64 (12)	C17—C18—H18B	109.5
C2—C1—C21	120.19 (12)	C17—C18—H18C	109.5
C14—C1—C21	120.05 (12)	H18A—C18—H18B	109.5
C1—C2—H2	118.9	H18A—C18—H18C	109.5
C1—C2—C3	122.19 (13)	H18B—C18—H18C	109.5
C3—C2—H2	118.9	C17—C19—H19A	109.5
C2—C3—C4	119.57 (12)	C17—C19—H19B	109.5
C2—C3—C22	120.87 (12)	C17—C19—H19C	109.5
C4—C3—C22	119.52 (12)	H19A—C19—H19B	109.5
C3—C4—C5	122.42 (12)	H19A—C19—H19C	109.5
C3—C4—C15	119.09 (12)	H19B—C19—H19C	109.5
C15—C4—C5	118.48 (12)	C17—C20—H20A	109.5
C4—C5—H5	119.3	C17—C20—H20B	109.5
C6—C5—C4	121.45 (13)	C17—C20—H20C	109.5
C6—C5—H5	119.3	H20A—C20—H20B	109.5
C5—C6—H6	119.3	H20A—C20—H20C	109.5
C5—C6—C7	121.41 (13)	H20B—C20—H20C	109.5
C7—C6—H6	119.3	O1—C21—C1	110.31 (11)
C8—C7—C6	122.16 (13)	O1—C21—H21A	109.6
C8—C7—C16	119.31 (13)	O1—C21—H21B	109.6
C16—C7—C6	118.52 (12)	C1—C21—H21A	109.6
C7—C8—H8	118.8	C1—C21—H21B	109.6
C9—C8—C7	122.39 (13)	H21A—C21—H21B	108.1
C9—C8—H8	118.8	O2—C22—C3	110.80 (11)
C8—C9—C17	119.52 (13)	O2—C22—H22A	109.5
C10—C9—C8	117.68 (13)	O2—C22—H22B	109.5
C10—C9—C17	122.80 (13)	C3—C22—H22A	109.5
C9—C10—H10	118.9	C3—C22—H22B	109.5
C9—C10—C11	122.28 (13)	H22A—C22—H22B	108.1
C11—C10—H10	118.9	O1—C23—H23A	110.1
C10—C11—C12	122.31 (12)	O1—C23—H23B	110.1
C10—C11—C16	119.37 (13)	O1—C23—C24	107.98 (15)
C16—C11—C12	118.32 (12)	H23A—C23—H23B	108.4
C11—C12—H12	119.2	C24—C23—H23A	110.1
C13—C12—C11	121.56 (13)	C24—C23—H23B	110.1
C13—C12—H12	119.2	C23—C24—H24A	109.5
C12—C13—H13	119.3	C23—C24—H24B	109.5
C12—C13—C14	121.50 (13)	C23—C24—H24C	109.5
C14—C13—H13	119.3	H24A—C24—H24B	109.5
C1—C14—C13	122.78 (12)	H24A—C24—H24C	109.5
C1—C14—C15	118.86 (12)	H24B—C24—H24C	109.5
C15—C14—C13	118.35 (12)	O2—C25—H25A	109.6
C4—C15—C14	120.64 (12)	O2—C25—H25B	109.6
C4—C15—C16	119.57 (12)	O2—C25—C26	110.12 (14)
C14—C15—C16	119.77 (12)	H25A—C25—H25B	108.1
C7—C16—C15	120.56 (12)	C26—C25—H25A	109.6
C11—C16—C7	118.94 (12)	C26—C25—H25B	109.6
C11—C16—C15	120.49 (12)	C25—C26—H26A	109.5
C9—C17—C20	112.03 (14)	C25—C26—H26B	109.5
C18—C17—C9	109.20 (13)	C25—C26—H26C	109.5
C18—C17—C20	108.61 (15)	H26A—C26—H26B	109.5
C19—C17—C9	109.68 (13)	H26A—C26—H26C	109.5
C19—C17—C18	110.15 (16)	H26B—C26—H26C	109.5
			
C1—C2—C3—C4	−0.15 (19)	C9—C10—C11—C12	−179.23 (13)
C1—C2—C3—C22	−178.02 (12)	C9—C10—C11—C16	0.1 (2)
C1—C14—C15—C4	0.52 (19)	C10—C9—C17—C18	−112.72 (17)
C1—C14—C15—C16	179.28 (11)	C10—C9—C17—C19	126.49 (18)
C2—C1—C14—C13	178.77 (12)	C10—C9—C17—C20	7.6 (2)
C2—C1—C14—C15	0.11 (19)	C10—C11—C12—C13	179.79 (13)
C2—C1—C21—O1	18.16 (18)	C10—C11—C16—C7	−0.90 (19)
C2—C3—C4—C5	−178.61 (12)	C10—C11—C16—C15	179.90 (11)
C2—C3—C4—C15	0.78 (18)	C11—C12—C13—C14	0.3 (2)
C2—C3—C22—O2	−7.55 (17)	C12—C11—C16—C7	178.45 (12)
C3—C4—C5—C6	179.82 (12)	C12—C11—C16—C15	−0.76 (19)
C3—C4—C15—C14	−0.97 (18)	C12—C13—C14—C1	−179.53 (13)
C3—C4—C15—C16	−179.72 (11)	C12—C13—C14—C15	−0.9 (2)
C4—C3—C22—O2	174.58 (11)	C13—C14—C15—C4	−178.20 (12)
C4—C5—C6—C7	0.0 (2)	C13—C14—C15—C16	0.55 (18)
C4—C15—C16—C7	−0.18 (18)	C14—C1—C2—C3	−0.3 (2)
C4—C15—C16—C11	179.01 (11)	C14—C1—C21—O1	−165.83 (12)
C5—C4—C15—C14	178.44 (12)	C14—C15—C16—C7	−178.95 (12)
C5—C4—C15—C16	−0.31 (18)	C14—C15—C16—C11	0.24 (19)
C5—C6—C7—C8	−179.81 (13)	C15—C4—C5—C6	0.43 (19)
C5—C6—C7—C16	−0.5 (2)	C16—C7—C8—C9	0.3 (2)
C6—C7—C8—C9	179.60 (13)	C16—C11—C12—C13	0.5 (2)
C6—C7—C16—C11	−178.64 (12)	C17—C9—C10—C11	−179.90 (13)
C6—C7—C16—C15	0.57 (19)	C21—O1—C23—C24	−173.86 (14)
C7—C8—C9—C10	−1.0 (2)	C21—C1—C2—C3	175.73 (12)
C7—C8—C9—C17	179.70 (13)	C21—C1—C14—C13	2.7 (2)
C8—C7—C16—C11	0.73 (19)	C21—C1—C14—C15	−175.92 (12)
C8—C7—C16—C15	179.94 (12)	C22—O2—C25—C26	174.09 (14)
C8—C9—C10—C11	0.9 (2)	C22—C3—C4—C5	−0.71 (19)
C8—C9—C17—C18	66.50 (18)	C22—C3—C4—C15	178.67 (11)
C8—C9—C17—C19	−54.3 (2)	C23—O1—C21—C1	−166.10 (12)
C8—C9—C17—C20	−173.13 (15)	C25—O2—C22—C3	172.25 (11)
